# Novel Antibacterial and Toughened Carbon-Fibre/Epoxy Composites by the Incorporation of TiO_2_ Nanoparticles Modified Electrospun Nanofibre Veils

**DOI:** 10.3390/polym11091524

**Published:** 2019-09-19

**Authors:** Cristina Monteserín, Miren Blanco, Nieves Murillo, Ana Pérez-Márquez, Jon Maudes, Jorge Gayoso, Jose Manuel Laza, Estíbaliz Hernáez, Estíbaliz Aranzabe, Jose Luis Vilas

**Affiliations:** 1 Unidad de Química de superficies y Nanotecnología, Fundación Tekniker, Iñaki Goenaga 5, 20600 Eibar, Spain; miren.blanco@tekniker.es (M.B.); estibaliz.aranzabe@tekniker.es (E.A.); 2 Division Industria y Transporte, TECNALIA, P Mikeletegi 7, E-20009 Donostia-San Sebastian, Spain; nieves.murillo@tecnalia.com (N.M.); ana.perez@tecnalia.com (A.P.-M.); jon.maudes@tecnalia.com (J.M.); jorge.gayoso@tecnalia.com (J.G.); 3 Grupo de Química Macromolecular (LABQUIMAC) Dpto. Química-Física, Facultad de Ciencia y Tecnología, Universidad del País Vasco (UPV/EHU), 48940 Leioa, Bizkaia, Spain; josemanuel.laza@ehu.eus (J.M.L.); estibaliz.hernaez@ehu.eus (E.H.); joseluis.vilas@ehu.eus (J.L.V.); 4 BCMaterials, Basque Center for Materials, Applications and Nanostructures, UPV/EHU Science Park, 48940 Leioa, Spain

**Keywords:** carbon-fibers, multifunctional composites, nanocomposites, fracture toughness

## Abstract

The inclusion of electrospun nanofiber veils was revealed as an effective method for enhancing the mechanical properties of fiber-reinforced epoxy resin composites. These veils will eventually allow the incorporation of nanomaterials not only for mechanical reinforcement but also in multifunctional applications. Therefore, this paper investigates the effect of electrospun nanofibrous veils made of polyamide 6 modified with TiO_2_ nanoparticles on the mechanical properties of a carbon-fiber/epoxy composite. The nanofibers were included in the carbon-fiber/epoxy composite as a single structure. The effect of positioning these veils in different composite positions was investigated. Compared to the reference, the use of unmodified and TiO_2_ modified veils increased the flexural stress at failure and the fracture toughness of composites. When TiO_2_ modified veils were incorporated, new antibacterial properties were achieved due to the photocatalytic properties of the veils, widening the application area of these composites.

## 1. Introduction

Carbon-fiber reinforced epoxy resin composites have gained popularity over the years as engineering materials mainly in the marine, aircraft, automotive, and civil engineering sectors. Among the reasons for their success should be highlighted their lightweight, high stiffness, and strength [1,2,3,4]. However, insufficient fracture toughness, due to the fragility of the epoxy matrix and poor delamination resistance which could contribute to sudden failure of the composite, affects the long-term reliability of thermosetting matrix composites [5,6].

In order to improve the delamination resistance of reinforced polymer composites, several approaches were tested. The most common way is the inclusion of some toughening elements or thermoplastic binders; nanoparticles, such as carbon nanotubes (CNTs) [7,8], nanoclays [9], and rubber/thermoplastic materials [10,11] are often incorporated in the matrix. Nanoparticles, as a result of their theoretical high stiffness and strength, might improve the fracture toughness and energy at crack growth initiation of epoxy systems [12,13,14]. However, the addition of toughener elements to the matrix resin could produce a decrease in mechanical properties such as compression and shear strength, also resulting in higher cost and weight. Even more, some disadvantages appear when adding CNTs and nanoclays, the main difficulty being obtaining a homogeneous dispersion of the nanoparticles in the resin [15]. At the same time, the increased viscosity arising from the addition of tougheners could also dramatically reduce the processing ability of matrix resin.

Recently, a method that could potentially overcome these drawbacks has raised interest. It involves the incorporation of thermoplastic electrospun nanofiber mats between the fiber reinforcing plies prior to processing. Reneker, Kim, and Dzenis [16,17] pioneered the work in this field to demonstrate toughness improvement; now, interlayer toughening is used increasingly in the composite industry [18,19,20]. Furthermore, due to the ease of embedding thermoplastic nanofibrous structures in resin and including them in the composite as a nanosized phase without an increase of resin viscosity, this is presented as a solution for the dispersion issue. Moreover, since their length is on the macro scale, no health hazards are associated with the production and handling of these nanofibers [21,22,23,24].

Recent literature indicates that nanofibers may contribute substantially to the ductility and fracture toughness of the composites [25,26,27]. The improvement is highly dependent on the type of thermoplastic and of their intrinsic properties [28]. The electrospinning technology also provides the possibility of including charges inside the nanofibers, which can improve their mechanical properties or give them different properties. The incorporation of metallic or electrically conductive carbonaceous nanoparticles such as silver nanowires or carbon nanofibers can also produce electrically conductive nanofiber veils. In recent years, major efforts have been dedicated to the manufacturing of antimicrobial membranes for ultrafiltration based on modified nanofibers. These antimicrobial functionalities can be introduced through different methods such as surface treatment or the incorporation of additives like antibiotics, biocides [29,30,31], or active agents. These agents include silver nanoparticles and nanoparticles of metals and metal oxides [32,33] such as Zn [34], Ti [28,35,36,37], Cu [38], Co [39], and combinations of Zn and Ti [40,41,42,43,44] among others [45,46]. The availability of functional nanofibers for the development of composite materials can be a great advantage in many sectors.

As mentioned above, the mechanical properties of thermoplastic electrospun nanofiber mats have an important effect on the mechanical properties of the reinforced composite. To increase the mechanical strength of thermoplastic electrospun nanofiber mats, several stronger nanoscale fillers were incorporated—such as graphene, carbon nanofibers (CNFs), nano clays, nano TiO_2_, and carbon nanotubes (CNTs) [47,48,49,50,51,52]. However, the obtainability of functional composites based on the incorporation of functional nanofibers was poorly studied.

In this paper, the effect of the inclusion of electrospun TiO_2_ modified polyamide 6 nanofiber veils on the mechanical and functional properties of carbon-fiber epoxy composites obtained by the infusion technique is explored. The nanofibrous veils are incorporated into the composite as single structures forming different configurations. The mechanical properties of the nanofiber veil-reinforced composites were investigated via flexural tests, dynamical mechanical analysis, and mode I and mode II fracture toughness tests against reference specimens. The crack propagation in the loaded sample was studied through scanning electron microscopy. Furthermore, new functionalities introduced by the presence of TiO_2_ nanoparticles in the nanofiber were determined by the analysis of the antibacterial activity of the composites.

## 2. Materials and Methods 

### 2.1. Materials

Diglycidyl ether of bisphenol A (DGEBA) was supplied by Momentive (Waterford, NY, USA), as Epikote 828, with an equivalent weight of 182–190 g/equivalent and a hydroxyl/epoxy ratio of 0.03. Epikote 828 crosslinking was obtained with 4,4′-diaminodiphenylmethane (DDM), supplied by Alfa Aesar (Lancashire, UK), which is characterized by a molecular weight of 198 g/mol and an amine equivalent weight of 49.5 g/mol. Electrospun nanofibers were prepared from PA6 Ultramid^®^ B24 N 03 pellets supplied by BASF SE (Ludwigshafen, Germany). For the manufacturing of the veils modified with nanoparticles, AEROXIDE^®^ TiO_2_ P25 nanoparticles supplied by Evonik (Essen, Germany) were used. These nanoparticles have a primary particle size of 21 nm and a specific surface area (BET) of approximately 50 m^2^/g. Due to the formation of aggregates and agglomerates between them, the density is approximately 130 g/cm^3^. They have a predominant structure of anatase, with an anatase/rutile weight ratio of approximately 80/20. A bidirectional carbon-fiber fabric formed by HT3k fibers (200 g/m^2^), supplied by SP Systems (Newport, UK) was employed for the production of the composite.

The development and characterization of electrospun PA6 and 25 wt.% TiO_2_ nanoparticle PA6 modified nanofiber veils was reported in previous work [53]. The characterization showed that TiO_2_ nanoparticles are widely dispersed on the surface of the nanofibers, providing the excellent photocatalytic activity of the veils under UV light irradiation (UV lamp Vl-6-L, from Vilber Lourmat (Collégien, France). Escherichia coli (E. coli) and other coliform bacteria were removed after 24 h of contact with the PA6/25 wt.% TiO_2_ electrospun nanofiber veils when irradiated with UV light.

### 2.2. Sample Preparation

**Figure 1:**
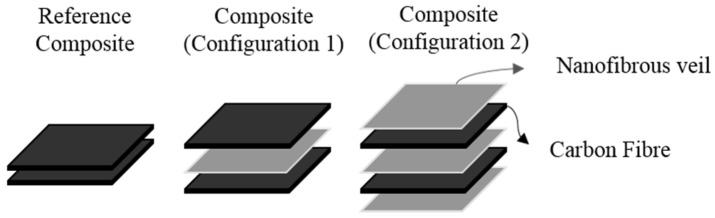
Configurations of the developed composites.

Theoretical composites with two carbon-fiber plies interlayered with PA6 and PA6/25 wt.% TiO_2_ electrospun nanofibrous veils were prepared. The veils were included only in the interlaminar region (Figure 1, configuration 1) or in interlaminar and also in the external layers of the composite (Figure 1, configuration 2). A composite with two carbon-fiber plies was used as a reference for comparison purposes. The composites were fabricated by vacuum infusion; where the carbon-fiber is impregnated by the liquid resin with the catalyst due to the vacuum effect by means of a vacuum bagging film. The infusion methodology is lineal to induce straight resin flow. DGEBA-DDM stoichiometric formulations were mixed for 10 min at 80 °C under stirring [54,55,56]. The 80 °C temperature was maintained during the vacuum infusion process through the whole laminate, and a slight increase in the infusion time of the resin for the composites with PA6 veils modified with 25 wt.% TiO_2_ was observed. Even though the veils were porous, the impregnation of all the layers of the composite required more processing time due to the increased areal weight in the veil modified with the nanoparticles—4.30 g/m^2^ for the TiO_2_ modified nanofibers versus 1.94 g/m^2^ for the unmodified nanofibers, calculated by weighing a piece of 10 cm × 10 cm of each veil. The same behavior was observed for the configuration with three veils. The composites were cured at 90 °C for 4 h, ensuring that the veils did not melt during curing. The incorporation of polyamide nanofiber veils did not affect the curing kinetics of the composite. The thermal characterization by differential scanning calorimetry (DSC) was performed in a DSC1 module from Mettler-Toledo (Giessen, Germany) can be found in the Supporting Information (Figure S1 and Table S1). The resulting composites were cut to the dimensions required for the different experimental analysis. The thicknesses of the fabricated composites with two carbon-fiber layers were between 0.6–0.7 mm. Following the AITM 1.0005 standard for the fracture test analysis (mode I, mode II), composites with 14 layers of carbon-fiber with one veil in the interlaminar region were fabricated by vacuum infusion technique, obtaining a thickness between 3.1–3.6 mm.

### 2.3. Characterization Techniques

The non-modified and 25 wt.% TiO_2_ modified PA6 electrospun nanofiber veils were characterized by Fourier transform infrared spectroscopy (FTIR) (JASCO, Easton, MD, USA) scanning electron microscopy (SEM) (Carl Zeiss Microscopy, LLC, Thornwood, NY, USA), thermogravimetric analysis (TGA) (TA instruments, New Castle, DE, USA), and weight measurements in a previous paper [53]. Moreover, the photocatalytic decomposition of an organic pollutant, Remazol Black B (C.I. Reactive Black 5), and against E. coli and other coliform bacteria (ISO 7704: 1985) under UV radiation was also evaluated.

In this work, they were characterized by differential scanning calorimetry (DSC) to determine the TiO_2_ nanoparticle influence on the crystallinity of the electrospun nanofibers. DSC measurements were performed with a Mettler-Toledo DSC1. Two successive temperature heating scans from 0 °C to 300 °C at 10 °C/min were performed. The mechanical properties of the composites were characterized by different techniques. Dynamic mechanical thermal analysis (DMTA) was performed using Polymer Laboratories Mark II DMTA equipment (Agilent Technologies, Santa Clara, CA, USA). All composite samples with PA6 and PA6/25 wt.% TiO_2_, as well as the reference system, were performed in dual cantilever mode from −50 °C to 200 °C at a heating rate of 2 °C/min, 64 µm strain, and 1 Hz. Rectangular samples (10 mm × 35 mm) were directly cut from the composite sheets. 

A three-point bending test was used to measure the flexural properties of the different composite samples using an electromechanical Instron 3369 machine with a load cell of 1 kN and a crosshead speed of 5 mm/min. Samples were cut to 60 mm × 20 mm, whilst avoiding the slipping of the specimens during the test. To calculate the flexural strength and deformation, the ISO 14125 methodology was followed. Mode I interlaminar fracture toughness studies were carried out using double cantilever beam (DCB) specimens following the AITM 1.0005 standard [57]. The DCB test specimens were prepared with dimensions of 250 mm × 25 mm and an initial crack length of 25 mm. Each experiment was repeated three times using a test speed of 10 mm/min; in each test, the specific thickness value was considered. Load, opening displacement, and crack length were recorded for the energy release rate (G_IC_, G_IIC_) calculation during the tests, as was explained in previous work [28]. The specimens employed in the analyses are the same three specimens that are tested in mode I. Scanning electron microscopy (SEM) and a Carl Zeiss SMT Ultra Gemini-II system were used to investigate the cross-sections after flexural analysis and Mode II fracture test of the composites.

A Microinstant^®^ Coliforms Chromogenic Agar (CCA) kit (Scharlab, Barcelona, Spain) was employed to perform the antibacterial tests and for the detection of E. coli and coliform bacteria. The water sample was filtered through a 0.45 μm pore size membrane filter, according to ISO 7704:1985. The membrane was deposited face up on a plate containing the CCA medium. The plate was incubated under incubation conditions: 18 h at 36 ± 2 °C, followed by 6 h if red or colorless colonies appear to include possible late reactions of ß-galactosidase or ß-glucuronidase. After the incubation period, colonies colored from salmon pink to red were counted as Coliform bacteria other than E. coli, and colonies colored from dark blue to violet as E. coli. The total coliform count corresponds to the sum of the salmon pink to red colonies and the dark blue to violet colonies. The antibacterial capability of the TiO_2_ modified PA6 nanofibers has previously been demonstrated [53].

## 3. Results and Discussion

### 3.1. Mechanical Characterization

To analyze the modified veil incorporation effect on the mechanical properties of composites, they were characterized by flexural, dynamic-mechanical, and fracture tests, along with SEM measurements.

**Figure 2:**
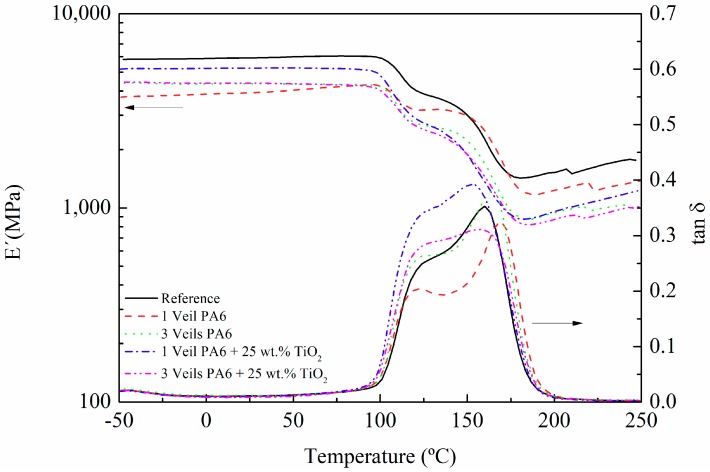
Storage modulus and tan δ of composites cured at a temperature of 90 °C.

DMTA is commonly used to measure viscoelastic properties like storage modulus (E′), which is related to the elastic stiffness; loss modulus (E″), which is related to the viscous response; and loss factor (tan δ), the ratio of the loss to the storage (i.e., the damping), which is related to the energy dissipation of the material. Figure 2 shows the storage modulus (E′) and tan δ change with respect to temperature of the composites reinforced with one and three nanofiber veils of PA6 and 25 wt.% TiO_2_ modified PA6. The value of E′ in the glassy zone (low temperatures) is lower for composites modified with veils. Nevertheless, it must be taken into account that the curing degree achieved was around 96% at this temperature (Table S1); therefore, the resin was not fully cured. In regard to tan δ, the first peak observed, above 100 °C, was related to the curing process of composites at 90 °C. As the temperature increased, the crosslinking continued, and a second peak was obtained above 150 °C, which corresponds to the Tg∞ of the system. The presence of the veils did not seem to have a clear improvement in the values of Tg∞, but the systems modified with TiO_2_ seemed to present a wider Tg∞ peak as a consequence of a more heterogeneous crosslinking due to the presence of nanoparticles.

The flexural strength and deformation of the carbon-fiber/epoxy composites with PA6 and 25 wt.% TiO_2_ modified PA6 electrospun nanofiber veils were carried out, and the main results are shown in Table 1 along with the values for reference composites. The experimental curves can be found in the Supporting Information (Figure S2). Unsurprisingly, the composites did not present delamination during testing. All composites with both PA6 and 25 wt.% TiO_2_ modified PA6 presented similar deformation values, but a clear increase in flexural strength against the reference composite was observed in all cases. For composites modified with one interlayered veil, an increase in flexural strength of 19.7% and 24.6% was observed for the composite with PA6 and 25 wt.% TiO_2_ modified PA6 electrospun nanofiber veils, respectively. The flexural strength increase is lower for the three veils specimens with and without titanium dioxide nanoparticles, indicating that veils, on the exterior faces, did not contribute to the improvement of the flexural mechanical properties.

**Figure 3:**
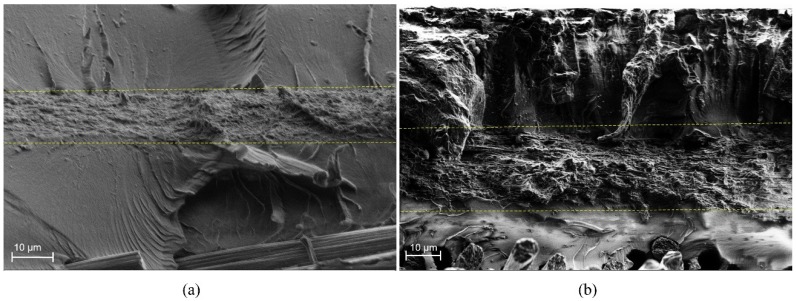
Scanning Electron Microscopy (SEM) images of (**a**) composite with PA6 veil and (**b**) composite with PA6 modified with 25 wt.% TiO_2_ veil. The dotted lines show the veil presence on the composites.

**Figure 4:**
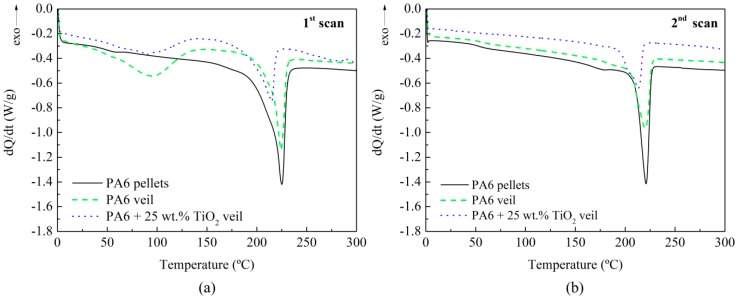
Dynamic differential scanning calorimetry (DSC) thermograms of the PA6 pellets, PA6 veil, and PA6 modified with 25 wt.% TiO_2_ veil; (**a**) first scan and (**b**) second scan.

After flexural tests, the fracture surfaces of the specimens were characterized by SEM. Figure 3 shows the interlaminar section of the (a) composite with PA6 veil and (b) composite with PA6 modified with 25 wt.% TiO_2_ veil. In both cases, fracture waves of the resin ended in the veil. The thicknesses of modified and non-modified veils were quite similar, 11 µm for unmodified and 20 µm for 25 wt.% TiO_2_ modified PA6 electrospun nanofiber veils, so the increase in flexural strength was due to the presence of the veil which prevented the propagation of the crack through the epoxydic matrix [58]. As reported in previous work [28], the veils on the external and internal faces of the composite did not seem to contribute to an improvement of the properties. For composites with one or three veils, the obtained flexural strength values indicated that the presence of TiO_2_ nanoparticles in the veils did not significantly affect the mechanical properties of the composites as initially expected [47,48,49,50,51,52]. On the one hand, TiO_2_ nanoparticles could affect crystallization kinetics of the PA6 polymer, reducing the mechanical properties of the nanofibers themselves. In order to check if the presence of TiO_2_ nanoparticles affected the crystallinity of PA6, modified and non-modified nanofiber veils were analyzed by DSC measurements. The PA6 pristine pellets used for obtaining these veils were also analyzed for a better result understanding. As can be observed in dynamic tests collected in Figure 4, the presence of a single melting peak almost between 224–225 °C in the first DSC scan of PA6 pellets reveals the presence of a higher proportion of α-form PA6 and a slight content of γ-form, whose melting temperatures appear at approximately 220 °C and 210 °C, respectively. For non-modified veils, apart from a slight peak at temperatures between 30–100 °C which was previously ascribed to the electrospinning process itself and is attributed to molecular rearrangement of PA6 chains within nanofibers [28,59,60], changes in PA6 nanofibers’ crystallinity have not been observed, and these nanofibers present a similar higher amount of α-form crystals (even if a lower crystallization degree was observed). However, for the TiO_2_ modified nanofibrous veils, the predominant crystalline phase observed was the γ-phase crystals of PA6. The γ-phase crystals, the predominant crystalline phase in the TiO_2_ modified veil, were a lower stable phase than the α-phase crystals (prevalent in the unmodified veil). This could have negatively affected the mechanical properties of the nanofibers and, therefore, we did not observe significant variations in mechanical properties.

On the other hand, the presence of the 25 wt.% TiO_2_ modified PA6 nanofiber veils in the composite could affect the crosslinking of the epoxy-amine matrix, as was shown in DMTA measurements (Figure 2), where a widening in the Tg_∞_ peak for the composites with the TiO_2_ modified nanofibers was observed, indicating a formation of a more heterogeneous structure.

**Figure 5:**
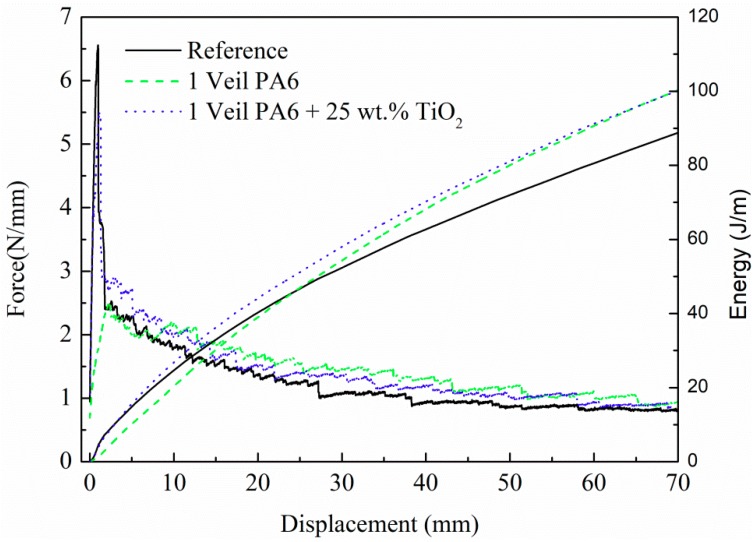
Mode I test results. Load and mechanical energy normalized to specimen width as a function of displacement.

Mode I and Mode II fracture toughness tests were conducted on composites with 14 layers of carbon-fiber fabric and an interlaminar veil. The experimental results of the G_IC_ tests-load-displacement and mechanical energy-displacement graphs-obtained for the specimens of composites with PA6 and 25 wt.% TiO_2_ modified PA6 nanofiber veils, as well as the results of the reference specimen, were presented in Figure 5, and the results were reported in Table 2. Both the force and the mechanical energy values were normalized to the specimen width. After an initial drop in load for low displacements in some of the composites, which was associated to the initiation of the crack propagation, composites with PA6 and 25 wt.% TiO_2_ modified PA6 nanofibrous veils present higher load values compared to the reference composite. Via the load-displacement diagram integration, the mechanical energy propagation was calculated, confirming the trend. The mechanical energy absorbed during the crack propagation test was improved by the presence of the veil at the crack opening interface. Also, the G_IC_ values for both composites with veils were higher than for the reference; approximately 20% and 14% for the composites with PA6 and 25 wt.% TiO_2_ modified PA6 nanofiber veils, respectively. However, the presence of the TiO_2_ nanoparticles did not seem to contribute to an enhancement of the improvement already obtained with the unmodified PA6 veil but, rather, the properties were maintained.

**Figure 6:**
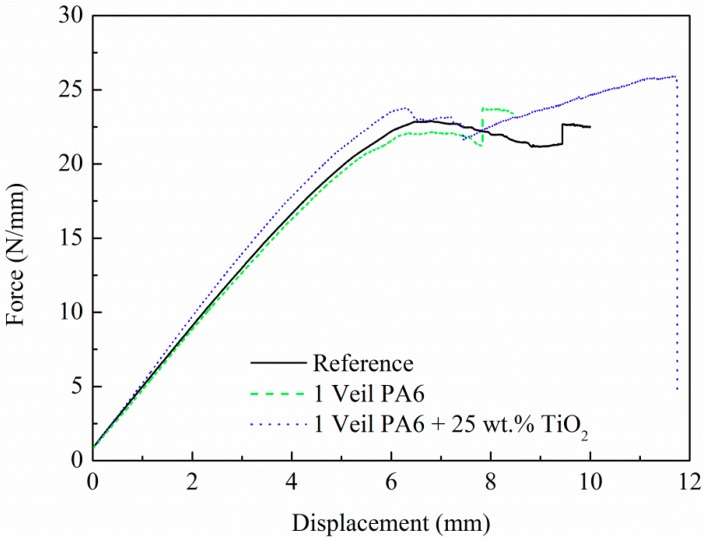
Mode II test results. Load normalized to specimen width as a function of displacement.

Figure 6 and Table 3 show the experimental results of the Mode II tests for composites with PA6 and 25 wt.% TiO_2_ modified PA6 nanofiber veils, along with results for reference composites. In Figure 6, the force values were normalized to specimen width. The veils do not influence the stability of the composites; thus, the initial linear parts of the curves were similar. However, the effect of the veil on the load capacity was visible as soon as the crack began to propagate. Comparing the G_IIC_ value obtained for the reference composite, a 4% of improvement was observed for the composite with the 25 wt.% TiO_2_ modified PA6 nanofiber veils compared to the reference composite. TiO_2_ nanoparticles added to the nanofiber polymer had a small influence on the fracture toughness of the composite, as the presence of the veil caused crack dispersion.

### 3.2. Antibacterial Behavior Characterization

**Figure 7:**
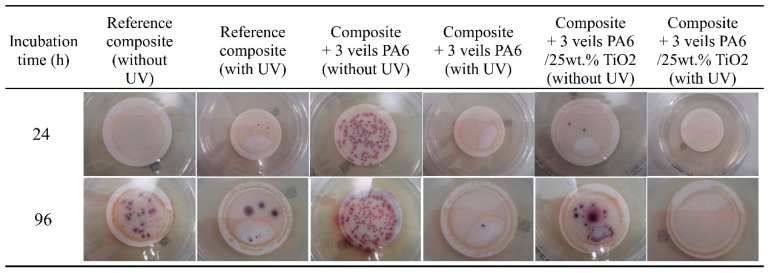
Antibacterial tests of composites with three veils of PA6, PA6 modified with 25% TiO_2_, and reference.

**Figure 8:**
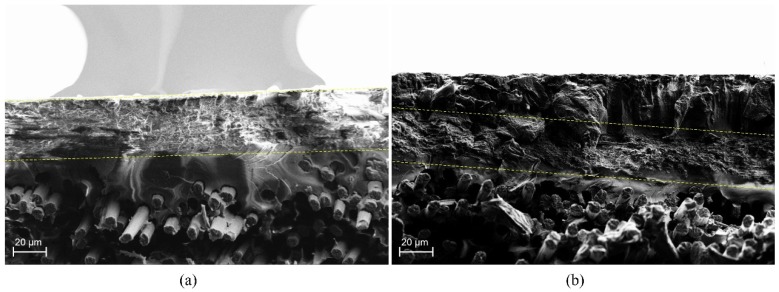
SEM images of the fracture surface of the composite specimens with PA6 modified with 25% TiO_2_ veils, (**a**) external part and (**b**) covered with resin. The dotted lines show the veil presence on the composites.

The efficiency of the 25 wt.% TiO_2_ modified PA6 nanofiber veils in E. coli and other coliform bacteria elimination was previously demonstrated [53]. Therefore, in the present work, whether the veil maintains this capacity once incorporated into the composite was evaluated. For this purpose, the configuration 2 (Figure 1) of the composites was selected due to the presence of the veils in the external layers of the composite, which were in contact with the inoculated water. An area of 4 × 4 cm was cut from the reference composite, as well as from the composites with three veils of PA6 and 25 wt.% TiO_2_ modified PA6 nanofiber veils. The same test procedure, as for the case of the veils, was followed. The composites were placed in beakers in contact with 100 mL of artificially inoculated water for 24 h. In some tests, the beakers were irradiated with UV light, and in others, they were kept in the dark. After this period, the water in contact with the composites was filtered to determine the presence of E. coli and other coliform bacteria (ISO 7704: 1985). The plate was incubated with the membrane for different durations (24 and 96 h) at 36 ± 2 °C. Figure 7 shows the final appearance of the membranes that were used to filter the water in contact with the composites after 24 h and 96 h of incubation. After 96 h of incubation in an oven, all the membranes which were in contact with composites in dark environments presented purple-colored colonies of bacteria, indicating the simultaneous action of the enzymes present in E. coli and coliform bacteria. When the study was done under UV radiation, a significant reduction in the bacterial concentration was observed. However, the membrane that was used to filter the water in contact with composites with 25 wt.% TiO_2_ modified PA6 nanofiber veils did not present any bacteria when the composite was irradiated with UV light. Therefore, the photocatalytic effect of the composites was confirmed and can be explained by the presence of part of the veils on the outer surface of the composites having direct contact with the bacteria. SEM images in Figure 8 confirm the presence of the TiO_2_ modified PA6 nanofiber in the external part of the composites or covered by around 20 μm of resin.

## 4. Conclusions

The present study aimed to investigate the effect of incorporating 25 wt.% TiO_2_ modified PA6 electrospun nanofiber veils on the mechanical and functional behavior of carbon-fiber/epoxy composites manufactured by a vacuum infusion process. 

The mechanical performance of the composites was evaluated by dynamic-mechanical tests, flexural, and fracture (mode I and II) tests. The incorporation of PA6 electrospun nanofiber veils increased the flexural mechanical properties. For composites modified with one interleafed veil, an increase in flexural strength of 19.7% and 24.6% was observed for the composite with PA6 and 25 wt.% TiO_2_ modified PA6 electrospun nanofiber veils, respectively. As the thickness of unmodified and modified veils was similar in the composites—11 µm for unmodified and 20 µm for 25 wt.% TiO_2_ modified PA6 electrospun nanofiber veils—the increase in flexural strength was ascribed to the crack deflection capability of the veils. When 25 wt.% TiO_2_ modified PA6 electrospun nanofiber veils were included in the composites, flexural strength values did not significantly increase compared to the results obtained for composites with unmodified veils. Initially, it was expected that TiO_2_ modified veils would increase flexural strength to a greater extent than unmodified veils. However, TiO_2_ nanoparticles could affect the crystallization of PA6, reducing the mechanical properties of the nanofibers themselves, as the γ-phase crystals (prevalent in the TiO_2_ modified veil) were less stable than the α-phase crystals (prevalent in the unmodified veil). Also, a negative effect of modified veils in the crosslinking structure of epoxy-amine network could contribute to this fact, as more heterogeneous networks were observed by DMTA for composites with 25 wt.% TiO_2_ modified PA6 electrospun nanofiber veils. For composites modified with three veils, the veils on the external faces of the conceptual composites did not contribute to an improvement of the flexural properties. Concerning the fracture behavior of composites, the G_IC_ values for composites with veils were higher than for the reference; approximately 20% and 14% for the composites with PA6 and 25 wt.% TiO_2_ modified PA6 nanofiber veils, respectively. However, the G_IIC_ values of composites with veils were similar to values for reference composites. In every case, the presence of the TiO_2_ nanoparticles had a small influence on the fracture toughness of the composites.

The incorporation of 25 wt.% TiO_2_ modified PA6 nanofiber veils with already proven antibacterial properties in the external surface of composites resulted in composites with antibacterial properties when irradiated with UV light due to the photocatalytic effect of TiO_2_ nanoparticles. This is due to the presence of the veils on some areas of the outer surface of the composites, as confirmed by SEM. The tested antibacterial capacity of composites against E. coli and other coliform bacteria could allow their use to be extended to applications where materials come into contact with microorganisms. For example, it could be used in marine applications, where the presence of fouling is a serious economic and safety problem; or in applications related to contact with people, such as the transport sector, sanitary sector, or food industry with applications in panels, containers, counters, or elements in contact with food in order to avoid the proliferation of dangerous bacteria.

## Figures and Tables

**Table 1:** Flexural strength (σ_max_) and deformation at break (δ_max_) for composites with PA6 [28] and 25 wt.% TiO_2_ modified PA6 nanofibrous veils.

Sample	σ_max_ (MPa)	Δσ_max_ (%)	δ_max_ (%)
Reference [28]	375.5 ± 33.2	-	2.2 ± 0.2
1veil PA6 [28]	449.5 ± 10.8	19.7	2.1 ± 0.0
3 veils PA6 [28]	415.4 ± 23.8	10.6	2.1 ± 0.2
1veil PA6 + 25 wt.% TiO_2_	468.1 ± 32.5	24.6	2.2 ± 0.2
3 veils PA6 + 25 wt.% TiO_2_	411.5 ± 25.4	9.6	2.1 ± 0.1

**Table 2:** Mode I results for composites with PA6 [28] and 25 wt.% TiO_2_ modified PA6 nanofibrous veils.

Sample	Energy (J/m)	ΔE%	G_IC_ (J/m^2^)	ΔG_IC_%
Reference [28]	62.7	-	389 ± 12.8	-
PA6 [28]	68.1	8.6	466 ± 72.9	20.0
PA6 + 25 wt.% TiO_2_	70.2	12.0	444 ± 46.1	14.0

**Table 3:** Mode II results for composites with PA6 [28] and 25 wt.% TiO_2_ modified PA6 nanofibrous veils.

Sample	G_IIC_ (J/m^2^)	ΔG_IIC_ (%)
Reference [28]	2536 ± 257	-
PA6 [28]	2544 ± 304	0.28
PA6 + 25 wt.% TiO_2_	2636 ± 124	3.95

## Supplementary Materials

The following are available online at https://www.mdpi.com/2073-4360/11/9/1524/s1, Figure S1: DSC thermographs of the composites with PA6 and PA6 modified with with 25 % TiO_2_ veils (a) 1st heating and (b) 2nd heating, Figure S2: Flexural strength and deformation curves, Table S1: DSC values of the composites with PA6 and PA6 modified with with 25 % TiO_2_ veils.
