# Supplementary material for: Novel Antibacterial and Toughened Carbon-Fibre/Epoxy Composites by the Incorporation of TiO2 Nanoparticles Modified Electrospun Nanofibre Veils
Source: Polymers (Basel). 2019 Sep 19;11(9):1524. doi: 10.3390/polym11091524 (PMC6780269; doi:10.3390/polym11091524)
Supplement: Supplementary file 1 [file polymers-11-01524-s001.pdf]

polymers

Supplementary Materials: Novel Antibacterial and Toughened Carbon Fibre/Epoxy Composites by the Incorporation of TiO_2 Nanoparticles Modified Electrospun Nanofibre Veils

Cristina Monteserín[1,*], Miren Blanco[1], Nieves Murillo[2], Ana Pérez-Márquez[2], Jon Maudes[2], Jorge Gayoso[2], Jose Manuel Laza[3], Estíbaliz Hernáez[3], Estíbaliz Aranzabe[1] and Jose LuisVilas[3,4]

Received: 22 July 2019; Accepted: 13 September 2019; Published: date

The thermal characterization of the curing kinetics has been done by means of differential scanning calorimetry (DSC). The composites were cured at 90 °C and subsequently characterized by DSC. Figure S1 shows the DSC thermograms of the composites with PA6 and PA6 veils modified with 25 % TiO_2. More specifically, Figure S1 (a) shows the residual reaction heat obtained in the first heating step and Figure S1 (b) shows the glass transition temperature of the fully cured material. Table 1 shows the residual heat obtained for each configuration of composite with PA6 veil and modified PA6 veil with 25 % TiO2 and the conversion degree for each sample calculated considering full conversion of the DGEBA-DDM system [1]. The residual heat values obtained for the composites with the modified veil are quite similar to those obtained for the unmodified veil, focusing on a range between 16–20 J/g, which results in a similar conversion degree of each composite system. It could be said that neither the incorporation of the PA6 veil nor the modified PA6 veil affects the curing of the composite material.

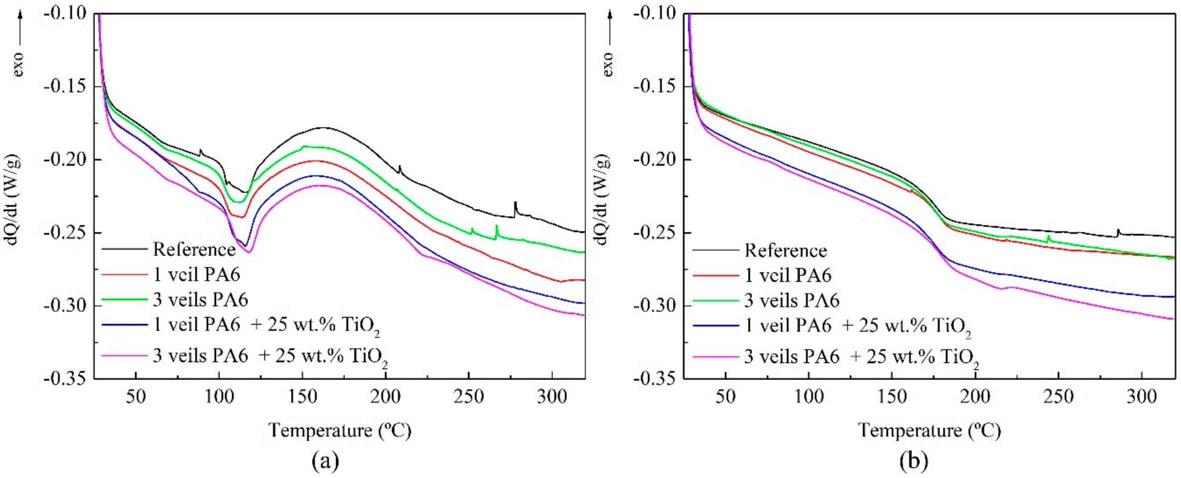

Figure S1. DSC thermographs of the composites with PA6 and PA6 modified with with 25 % TiO2 veils (**a**) 1st heating and (**b**) 2nd heating.

Table S1. DSC values of the composites with PA6 and PA6 modified with with 25 % TiO2 veils.

Material	ΔH_{res} (J/g)	α (%)
Referencia	18.8	96.4
1 Velo PA6 Ultramid	20.3	96.2
3 Velos PA6 Ultramid	16.9	96.8

1 Velo PA6 Ultramid + 25 % TiO_2	20.5	96.1
3 Velos PA6 Ultramid + 25 % TiO_2	21.5	95.9

Figure S2, shows the flexural strength and deformation of the carbon fibre/epoxy composites with PA6 and 25 wt.% of TiO_2 modified PA6 electrospun nanofibre veils, along with the values for reference composites

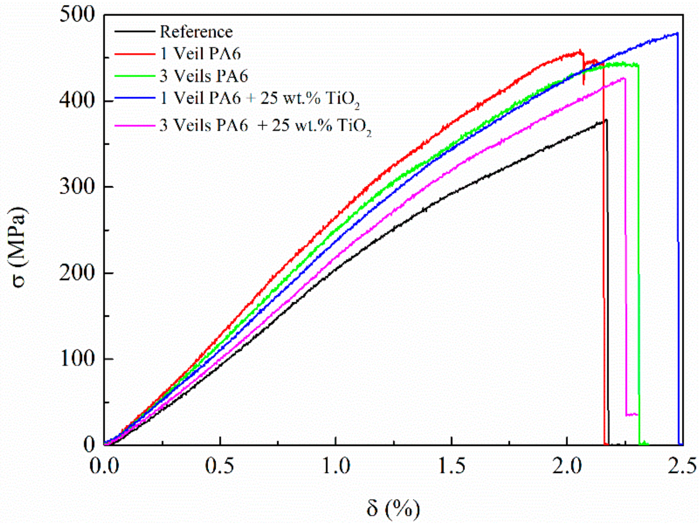

Figure S2. Flexural strength and deformation curves.

References

1. Monteserín, C.; Blanco, M.; Laza, J.M.; Aranzabe, E.; Vilas, J.L. Thickness effect on the generation of temperature and curing degree gradients in epoxy–amine thermoset systems. *J. Therm. Anal. Calorim*. **2018**, *132*, 1867–1881.

© 2019 by the authors. Submitted for possible open access publication under the terms and conditions of the Creative Commons Attribution (CC BY) license (http://creativecommons.org/licenses/by/4.0/).
